# Gene Therapy for Parkinson’s Disease Associated with *GBA1* Mutations

**DOI:** 10.3233/JPD-212739

**Published:** 2021-09-22

**Authors:** Asa Abeliovich, Franz Hefti, Jeffrey Sevigny

**Affiliations:** Prevail Therapeutics, A Wholly-Owned Subsidiary of Eli Lilly and Company, New York, NY, USA

**Keywords:** Gene therapy, GBA1, glucocerebrosidase, Gaucher, AAV9

## Abstract

Human genetic studies as well as studies in animal models indicate that lysosomal dysfunction plays a key role in the pathogenesis of Parkinson’s disease. Among the lysosomal genes involved, *GBA1* has the largest impact on Parkinson’s disease risk. Deficiency in the *GBA1* encoded enzyme glucocerebrosidase (GCase) leads to the accumulation of the GCase glycolipid substrates glucosylceramide and glucosylsphingosine and ultimately results in toxicity and inflammation and negatively affect many clinical aspects of Parkinson’s disease, including disease risk, the severity of presentation, age of onset, and likelihood of progression to dementia. These findings support the view that re-establishing normal levels of GCase enzyme activity may reduce the progression of Parkinson’s disease in patients carrying *GBA1* mutations. Studies in mouse models indicate that PR001, a AAV9 vector-based gene therapy designed to deliver a functional *GBA1* gene to the brain, suggest that this therapeutic approach may slow or stop disease progression. PR001 is currently being evaluated in clinical trials with Parkinson’s disease patients carrying *GBA1* mutations.

## INTRODUCTION

Parkinson’s disease (PD) is a disease affecting the peripheral and central nervous systems that results in the dysfunction and loss of certain neuronal populations and clinically manifests with a plethora of motor and a non-motor symptoms. The lysosome is responsible for degradation and recycling of various proteins and other cellular components, and it is now widely recognized that lysosomal dysfunction plays a role in the pathogenesis of PD. Lysosomal dysfunction underlies the pathological accumulation of α-synuclein, a major component of the Lewy body intracellular aggregates, and leads to toxicity and inflammation. Human genetic studies have identified over 40 potentially causative and risk associated genes for Parkinson’s disease, and many of these genes are implicated in lysosomal function or lysosomal trafficking, indicating that lysosome dysfunction is a common denominator underlying Parkinson’s disease pathology. Of these, *GBA1*, which encodes the lysosomal enzyme glucocerebrosidase (GCase), stands out due to the relatively high prevalence of pathological mutations, the effect such mutations on the risk of developing PD, and their aggravating effect on clinical course of the disease. Animal model studies support the hypothesis that increasing GCase levels in PD patients’ CNS is a promising therapeutic approach to slow or stop disease progression.

## PARKINSON’S DISEASE ASSOCIATED WITH THE *GBA1* GENE

Glucosylceramidase (GCase) is a lysosomal enzyme encoded by the *GBA1* gene and catalyzes the conversion of glucosylceramide (GluCer) into glucose and ceramide. GCase deficiency leads to the accumulation of GluCer, glucosylsphingosine (GluSph), and other glycolipid derangements, resulting in toxicity and inflammation [1–3]. GCase may play additional roles in lysosome function and protein homeostasis, and has been hypothesized to directly or indirectly impact α-synuclein aggregation [4–7]. Approximately 300 *GBA1* mutations have been reported [8]. These mutations lead to various degrees of loss of function of GCase activity, and this is reflected in the variable extent and severity of clinical manifestations ranging from asymptomatic carriage to perinatal hydrops fatalis caused by Gaucher disease [2, 9, 10]. Gaucher disease has traditionally been subdivided into three phenotypes, herein referred to as GD1, GD2, and GD3. GD1 is considered to be the non-neuronopathic form, classically manifesting with hepatosplenomegaly and attendant sequelae, although GD1 patients are at high risk of developing PD as described below. GD2 and GD3 are the neuronopathic forms, having neurological in additional to the peripheral manifestations that accompany GD1 [11]. Of the two neuronopathic forms, GD2 is the more severe form, typically presenting in infanthood with multiple bulbar signs and symptoms including swallowing difficulty and supranuclear gaze palsy, opisthotonos, spasticity, epilepsy, and failure to thrive. Death typically occurs before age 2 [12]. GD3 usually presents in early childhood or adolescence with horizontal supranuclear gaze palsy and is often accompanied with other neurological manifestations including ataxia, myoclonic epilepsy, and learning disabilities [13, 14]. The clinical course tends to be slow. The distinction between GD2 and GD3 can be challenging, suggesting that the various forms of GD represents a phenotypic continuum [15].

*GBA1* mutation(s) also increases one’s risk of developing PD, from 5× for mono-allelic carriers to up to 20× for bi-allelic carriers, as well as of related neurodegenerative disorders such as Lewy Body Dementia or Parkinson’s Disease Dementia [16–18]. It is estimated that 5% to 25% of patients with PD carry *GBA1* mutation(s), and up to 30% of carriers will develop PD by age 80 [19, 20]. In light of the relatively large effect of *GBA1* mutations on PD risk and the relatively high prevalence of these mutations, *GBA1* mutations are considered to be the most important genetic cause of PD.

The extent and severity of the diseases caused by GBA1 mutations relate to the extent of the GCase deficiency. For example, GCase activity is reduced by approximately 20% to 50% in PD-GBA, often greater than 90% in neuropathic forms of GD, to virtually 100% in hydrops fetalis [2, 21–23]. The risk of developing PD follows the same “dose effect” principle: those with more severe *GBA1* mutations (e.g., “neuronopathic” mutations) and/or biallelic carriage generally have lower GCase activity and are at higher risk of PD relative to patients with less severe mutations or mutations in only 1 chromosomal copy [22, 24–27].

The spectrum of the clinical manifestations of PD-GBA is similar to idiopathic PD, but PD-GBA tends to manifest at an earlier age, progress more rapidly, and be accompanied by cognitive impairment, and therefore is considered a more aggressive form of PD [19, 28–31]. Even in the absence of specific *GBA1* mutations, reduced GCase activity in patients with idiopathic PD has been reported by multiple groups, further lending support to the hypothesis that reduced GCase is central to the development of PD [22, 32–34]. The observation of a genetic “dose-effect” of the level of GCase reduction correlating with the severity of disease, strongly supports the notion that an increased level of GCase would be beneficial in these individuals.

**Fig. 1:**
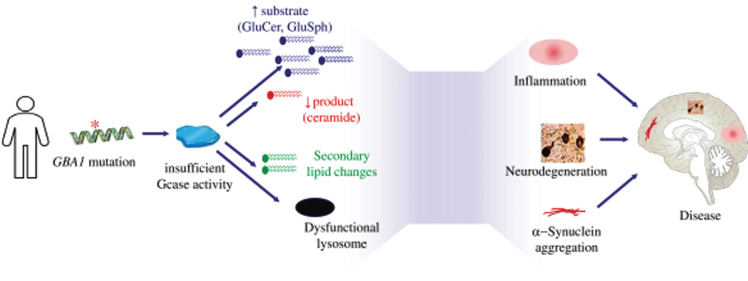
Pathophysiology of Parkinson’s disease associated which *GBA1* mutations.

The pathophysiology of PD-GBA is partially understood. Reduced level of GCase activity in GBA-associated Parkinson (PD-GBA) patients is believed to lead to accumulation of glycolipid substrates including GluCer and GluSph, as well as altered production of ceramide and secondary changes in other lipids. The glycolipid substrate accumulation is toxic and pro-inflammatory, leading to lysosomal dysfunction. There is also evidence that accumulation of these glycolipids may impact the structure and aggregation of α-synuclein in cells. The reduction in ceramide has also been linked to neurodegeneration and α-synuclein pathology in model systems and has been observed in Parkinson’s disease brain. In animal models, GluCer accumulation correlates with increased aggregation of phosphorylated α-synuclein protein and aggregates [35, 36]. The pathophysiology of PD associated with GBA mutations is illustrated in Fig. 1.

## GENE THERAPY TRANSDUCING *GBA1* IN THE BRAIN

Delivering a normal *GBA1* gene using an adeno-associated virus (AAV) vector to a variety of animal models of PD or PD-GBA, including various genetic models with α-synuclein or GBA1 gene mutations, has been shown to be efficacious by multiple independent investigators [37–41]. Most of the these *in vivo* studies delivered the AAV-GBA1 vector directly into the rodent CNS, although in at least two examples, intravenous delivery was used [42]. Increasing GCase levels and activity through the delivery of a GBA1 vector has been reported to reduce inflammation as well as the accumulation of aggregated α-synuclein in these multiple independent models of PD. Additional studies have used non-genetic approaches to increase GCase activity in rodent PD models and reported efficacy in the reduction of pathological features [43–45].

Based on the consideration above, we have pursued the development of a gene therapy to deliver a functional *GBA1* gene to the CNS for PD-GBA patients. Our investigational drug, called PR001, represents the first clinic-staged experimental gene therapy for these patients. We selected the adeno-associated virus serotype 9 (AAV9) as the vector for a codon-optimized plasmid encoding a wild-type human *GBA1* gene. AAV9 was chosen since it has demonstrated efficient brain transduction and multiple clinical experiences with this vector has shown a favorable safety profile in humans [46]. PR001 contains elements to constitutively express *GBA1* under the control of the CMV enhancer and CBA promoter using a codon optimized coding sequence for *GBA1*.

PR001 efficacy was examined in established models of GCase deficiency that display phenotypic characteristics consistent with GD and PD-GBA (the preclinical findings obtained with PR001 were reported at scientific conferences [50, 51]). First, the CBE: conduritol-β-epoxide (CBE) mouse model is a chemical model in which a pharmacological inhibition of GCase activity is achieved using a selective and irreversible covalent competitive inhibitor of GCase, leading to glycolipid (GluCer and GluSph) accumulation, neuropathological changes including astrogliosis and microgliosis, and motor behavior eficits [47–49]. Second, the 4L/PS-NA mouse model is a genetic model that harbors mutations in *Gba1* (V394L) and in the *PSAP* gene that encodes saposin C, an activator of GCase, and which displays a severe reduction in GCase activity, an accumulation of glycolipid substrates, and motor behavior deficits [52, 53]. In addition, the 4L/PS-NA mouse model displays accumulation of *α*-synuclein.

In the CBE model, intraventricular PR001 treatment resulted in increased GCase expression, elevation of GCase enzyme activity, reduction of the accumulation of glycolipid substrates of GCase, correction of neuroinflammation, and behavioral improvements. Broad vector genome biodistribution of PR001 was seen in the CNS and peripheral organs, including the liver, spleen and lung. In the 4L/PS-NA model, PR001 treatment resulted in sustained GCase expression and suppression of glycolipid accumulation over 6 months and amelioration of the behavioral deficits. In our studies, insoluble *α*-synuclein levels in the cerebral cortex were nonsignificantly increased in the 4L/PS-NA relative to control mice, as reported in published studies and PR001 treatment reversed such accumulation, consistent with our *in vitro* analyses in cell cultures [6, 52, 54].

PR001 safety was evaluated in both mouse models and no adverse histopathologic findings or evidence of toxicity due to treatment was observed. Biodistribution and toxicology studies were performed in nonhuman primates. Broad distribution of PR001 vector and significant elevation of GCase protein levels were observed in the brain. There were no adverse findings or evidence of toxicity due to PR001.

PR001 is currently in early clinical trials in PD-GBA patients (PROPEL) and in children with GD2 (PROVIDE) [55, 56; http://www.clinicaltrials.gov]. PR001 is administered by a single injection into the cisterna magna of the intrathecal space. Animal studies have demonstrated that, by obviating the need to cross the blood brain barrier, intrathecal delivery results in efficient CNS gene transfer [57–60]. Lumbar puncture is the most common method for accessing CSF. However, delivery of an AAV9 vector into the CSF via a lumbar puncture was found to be at least 10-fold less effective at transducing cells of the brain and spinal cord compared to injection of the vector more superiorly at the level of the cisterna magna [55, 56] Adult rhesus macaques injected with the clinical candidate vector via suboccipital puncture into the cisterna magna exhibited motor neuron transduction at all levels of the spinal cord. In contrast, animals receiving vector injection via lumbar puncture showed substantially lower transduction at all levels of the spinal cord. This study illustrated the potential of delivering vector at the level of the cisterna magna, and supported the selection of suboccipital puncture as the clinical route of administration.

## CONCLUSIONS

Human genetic studies strongly support a causative role of GCase deficiency in PD-GBA. Consistent with this, studies in animal models have demonstrated that increasing GCase activity by the delivery of the wild-type *GBA1* gene can be efficacious. Clinical studies in PD-GBA and GD2 are now ongoing to investigate the hypothesis that increasing GCase slows or stops progression of the disease.

## CONFLICT OF INTEREST

All authors are or have been employees of Prevail Therapeutics – a wholly owned subsidiary of Eli Lilly and Company.
